# DNA methylation-mediated silencing of HNF1B promotes bladder cancer progression

**DOI:** 10.1186/s13148-026-02079-z

**Published:** 2026-02-21

**Authors:** Cong Luo, Xiaogen Kuang, Yuanqing Dai, Zhiyong Cai, Jiao Hu, Ping Liu, Jinbo Chen

**Affiliations:** 1 Department of Anesthesiology, Xiangya Hospital, Central South University, Changsha, China; 2 Department of Urology, Xiangya Hospital, Central South University, Changsha, China; 3 Department of Urology, The First Affiliated Hospital, Hengyang Medical School, University of South China, Hengyang, Hunan China; 4 Department of Radiation Oncology and Hunan Key Laboratory of Translational Radiation Oncology, The Affiliated Cancer Hospital of Xiangya School of Medicine, Central South University/Hunan Cancer Hospital, Changsha, China; 5 National Clinical Research Center for Geriatric Disorders, Xiangya Hospital, Central South University, Changsha, China

**Keywords:** HNF1B, DNA methylation, Bladder cancer, MAPK pathway

## Abstract

**Background:**

Hepatocyte nuclear factor 1 homeobox B (HNF1B), a developmentally crucial transcription factor, demonstrates tumor-suppressive functions in several cancers, with epigenetic silencing being a known mechanism, as seen in malignancies like ovarian and prostate carcinoma. Its functional significance in bladder cancer pathogenesis is far less clear.

**Results:**

Our analysis of clinical cohorts identified low HNF1B expression as an independent prognostic factor that is significantly associated with adverse clinicopathological characteristics and poorer survival in bladder cancer patients. Functionally, knockdown of HNF1B promoted bladder cancer cell proliferation, migration, and invasion, whereas its overexpression suppressed these malignant phenotypes in vitro and attenuated tumor growth in vivo. Mechanistically, HNF1B silencing was primarily mediated by promoter hypermethylation. Furthermore, we demonstrated that HNF1B exerts its tumor-suppressive roles by concurrently inhibiting the mitogen-activated protein kinase (MAPK) signaling pathway and the epithelial–mesenchymal transition (EMT) process.

**Conclusion:**

Our study unveils a novel epigenetic mechanism in bladder cancer, whereby promoter hypermethylation silences HNF1B, thereby promoting tumor progression through activation of the MAPK pathway. These findings establishe HNF1B status as a potential biomarker for stratifying patients who may benefit from targeted therapies against this pathway.

**Supplementary Information:**

The online version contains supplementary material available at 10.1186/s13148-026-02079-z.

## Background

Bladder cancer (BLCA) is a prevalent genitourinary malignancy with a rising incidence rate [1]. A significant proportion of patients present with muscle-invasive disease, known for its aggressive features of progression, metastasis, and recurrence. Recent advancements in treatment strategies, such as immunotherapy and targeted therapy, have improved outcomes for BLCA patients[2]. Nevertheless, the response rate in advanced BLCA patients remains poor, largely due to drug resistance and treatment-related toxicities [3]. Therefore, it is crucial to investigate the molecular mechanisms driving BLCA progression. This will not only help identify novel therapeutic targets but also pave the way for precision medicine in BLCA.

HNF1B is a transcription factor primarily pivotal for developing organs originating from the primitive endoderm, including the pancreas, liver, and kidneys. As a member of the hepatocyte nuclear factor family, it plays a fundamental role in early tissue patterning and differentiation. Beyond its essential functions in fetal development, its expression persists in adult tissues, including the genitourinary tract, as supported by data from the Human Protein Atlas (HPA) (Fig. S1C). In murine models, genetic ablation of HNF1B specifically in the ureteric bud leads to severe renal developmental defects through disruption of branching morphogenesis and impairment of the mesenchymal-to-epithelial transition [4, 5]. In humans, mutations in HNF1B cause a spectrum of congenital renal anomalies, including structural abnormalities (*e.g.*, cysts, dysplasia) to functional impairments (*e.g.*, hypomagnesemia, progressive renal decline) [6].

Beyond its well-established roles in development and benign disease, HNF1B plays a complex role in cancer pathogenesis. Genomic studies have linked specific HNF1B polymorphisms to increased susceptibility to malignancies such as prostate and pancreatic cancer, revealing its broad role in cancer predisposition [7–12]. HNF1B can function as either a tumor suppressor or an oncogene, depending on the tissue type. For instance, in pancreatic ductal adenocarcinoma and prostate cancer, it exerts tumor-suppressive effects via inhibiting the EMT process [13, 14]. Conversely, in ovarian clear cell carcinoma, it acts as an oncogenic driver that orchestrates metabolic reprogramming to establish a pro-survival niche under stress conditions[15].

However, HNF1B's functional significance in BLCA remains poorly understood. Current evidence is limited to arsenic-induced bladder carcinogenesis models, where it acts as a stem cell activator facilitating malignant transformation [16]. This substantial gap underscores the need investigate HNF1B's pathophysiological role in BLCA. Our analyses have revealed that HNF1B serves as a favorable prognostic factor in BLCA patients. Herein, we aim to elucidate the biological functions and regulatory mechanisms of HNF1B in BLCA, from cellular and animal models to clinical correlates. Our findings not only advance the understanding of BLCA progression but also point to novel therapeutic avenues.

## Methods

### Data collection and processing

In June 2024, we obtained two publicly available BLCA datasets: The Cancer Genome Atlas Bladder Urothelial Carcinoma (TCGA-BLCA) cohort (n = 408) and the Xiangya cohort (n = 57, accession number GSE188715). For the TCGA-BLCA cohort, samples with missing prognostic information were excluded from analysis. The clinicopathological characteristics of the Xiangya cohort are provided in Table S1.

We employed the R package (BLCAsubtyping) to perform Lund molecular subtyping on the acquired cohort dataset [17]. Multivariable Cox regression analysis (survival package) was performed to evaluate the association of predictor variables with patient survival.

The Gene Set Cancer Analysis (GSCA) platform integrates multi-omics data from TCGA to systematically correlate HNF1B expression with DNA methylation (at specific CpG sites), somatic mutations, and copy number variation (CNV) profiles. All GSCA analyses were performed with default parameters (http://bioinfo.life.hust.edu.cn/GSCA/#/).

### RNA sequencing and data processing

Total RNA was extracted from BLCA cell lines following a standard protocol. Library preparation and sequencing were performed by Novogene Co., Ltd. Raw sequencing data were processed by converting FPKM (Fragments Per Kilobase of transcript per Million mapped reads) to TPM (Transcripts Per Kilobase Million) values for analysis. For downstream analysis, data were cleaned and normalized by: (1) removing genes with zero expression in > 80% of samples or > 50% missing values, and (2) applying a log2(TPM + 1) transformation to all expression values.

### Differential expression analysis and enrichment analysis

Differential expression analysis was performed using the R package limma (v3.40.6) to identify differentially expressed genes (DEGs) [18]. For functional enrichment, we used the Hallmark gene sets (org.Hs.eg.db, v3.1.0) as the reference background. Gene mapping and enrichment were conducted with clusterProfiler (v3.14.3). Gene Set Enrichment Analysis (GSEA) was carried out using GSEA software (v3.0; Broad Institute) with the Hallmark and KEGG pathway gene sets from the Molecular Signatures Database (MSigDB) to evaluate relevant pathways and mechanisms.

### Clinical samples

In addition to the Xiangya transcriptomic cohort used for bioinformatic analysis, this study utilized two independently collected patient cohorts for experimental validation. The first cohort (pyrosequencing cohort) comprised 22 primary BLCA tissues obtained with informed consent from Xiangya Hospital, Central South University, between June and December 2022. All cases were histopathologically confirmed in accordance with World Health Organization criteria. Clinical and pathological data for this cohort were collected retrospectively. This cohort was used to assess the epigenetic regulation of HNF1B. The second cohort (tissue microarray cohort) consisted of an independent set of 60 BLCA specimens. After quality control excluding 7 samples with insufficient cellularity (< 10,000 cells), 53 qualified samples in the tissue microarray were used to investigate the relationship between HNF1B expression and the MAPK pathway activity via multiplex immunofluorescence staining. The study was conducted in accordance with the Declaration of Helsinki.

### Cell culture and functional analyses

The BLCA cell lines used (T24, 5637, UM-UC-3, RT4, and RT112) were cultured in their recommended media: DMEM for T24 and UM-UC-3; RPMI-1640 for 5637 and RT112; and McCoy's 5A for RT4 (Table S2). All media were supplemented with 10% fetal bovine serum (FBS) and 1% penicillin–streptomycin.

Cell proliferation was assessed using cell counting kit-8 (CCK-8) and colony formation assays. The CCK-8 assay was performed following the manufacturer's instructions. For colony formation, cells were seeded in 6-well plates at 1,000 cells/well. After 10 days, colonies were fixed, stained, and counted.

Cell migration was assessed by wound healing and transwell assays. For wound healing, confluent monolayers were scratched with a 200-μl pipette tip and wound closure was measured after 24 h. For transwell assays, cells in serum-free medium were seeded into the upper chamber (Matrigel-coated for invasion assays), with 10% FBS medium as a chemoattractant below. after 48 h, transmigrated cells were fixed, stained with crystal violet, and quantified.

### Lentivirus transduction

Lentiviral particles for HNF1B overexpression, knockdown (Table S3), and their respective negative controls (empty vector and scrambled shRNA) were purchased from GeneChem Co., Ltd. (Shanghai, China). Cells were transduced according to the manufacturer's protocol. Green fluorescent protein (GFP) expression was assessed at 72 h to confirm transduction, and puromycin was then used to select stable cell lines.

### Quantitative reverse transcription PCR (qRT-PCR) and Western blotting

Total RNA was extracted using the SteadyPure Universal RNA Extraction Kit and reverse-transcribed with the ABScript III RT Master Mix for qRT-PCR. qRT-PCR was performed on a QuantStudio 7 Real-Time system using the Genious 2X SYBR Green qPCR Mix, with primers listed in Table S3. Total protein was extracted using RIPA lysis buffer, and protein concentration was measured using a BCA assay kit. Subsequently, Western blotting was conducted following standard protocols. The antibodies used are summarized in Table S4.

### Xenograft mouse model

Female BALB/c nude mice, aged 6–8 weeks, were obtained from Hunan SJA Laboratory Animal Co., Ltd. To assess the in vivo impact of HNF1B on BLCA growth, 5637 cells stably expressing either the empty vector (control) or HNF1B (5 × 10⁶ cells per mouse) were subcutaneously injected into the flanks of nude mice to establish xenograft models. The tumor size and mouse weight were assessed every seven days. Tumor volume was calculated using the formula V = L × W^2^/2, where L is the length and W is the width.

### Immunohistochemical (IHC) and multiplex immunofluorescence staining

IHC was performed on two types of BLCA tissues: clinical specimens and mouse xenograft tumors. Sections were incubated with primary antibodies (HNF1B or Ki67) at 4 °C overnight, followed by an HRP-conjugated secondary antibody. Staining was visualized using a Nikon microscope. For quantification, five non-overlapping fields per sample were randomly captured with a Leica QWin Plus system. Positive staining for Ki67 and HNF1B was defined by brown-yellow nuclear granules, and the percentage of positive cells was calculated as (positive cells/total cells) × 100%.

Multiplex immunofluorescence staining was performed using a TSA fluorescence kit (Affibody Biotechnology Co., Ltd) according to the manufacturer's protocol. rimary antibodies against p-MEK1/2 (Ser218/222), p-ERK1/2 (Thr202/Tyr204), HNF1B, MEK1/2, and ERK1/2 were applied (Table S4), followed by nuclear counterstaining with DAPI. Whole-slide imaging was conducted using a Zeiss Axioscan 7 system. The tissue regions of interest were selected, and the target objects were quantitatively measured using the fluorescence co-expression module in Visiopharm software.

### DNA methylation detection

Methylation of CpG islands in the HNF1B promoter was analyzed using bisulfite sequencing PCR (BSP) in BLCA cell lines according to established protocols [19]. The detailed genomic coordinates and specific information for each analyzed CpG site are provided in Table S5. In primary BLCA tissues, genomic DNA was bisulfite-converted using an EpiTect Bisulfite kit (Qiagen). Quantitative methylation analysis of CpG sites within the HNF1B promoter was then performed by pyrosequencing on the PCR-amplified target regions. The primers associated with BSP and pyrosequencing are presented in Table S3. For both BSP and pyrosequencing assays, the promoter methylation status for each cell line or sample was represented by the average methylation rate across all CpG sites analyzed.

### Cell treatment

At 50% confluence, 5637 and UM-UC-3 cells were treated with 5-Azacytidine (5-AzaC) at concentrations of 0 μM, 5 μM, 10 μM, and 20 μM for 72 h. To establish optimal trametinib doses, dose–response curves were generated in T24 and 5637 cells. Non-cytotoxic, effective concentrations were then selected for subsequent experiments. Lentivirus-transfected cells were treated with trametinib at optimized concentrations or with DMSO vehicle. Cell viability was assessed by CCK-8 assay. After 4 days of treatment, protein lysates were harvested and analyzed by Western blotting for MAPK pathway components.

### Statistical analyses

All statistical analyses were performed using GraphPad Prism (Version 5.0). Data are presented as mean ± standard deviation (M ± SD). Comparisons between groups were made using an unpaired two-tailed Student's t-test or one-way ANOVA with Tukey's post hoc test, as appropriate. A *P*-value of < 0.05 was considered statistically significant.

## Results

### Low HNF1B expression correlates with adverse prognosis in BLCA patients

**Fig. 1:**
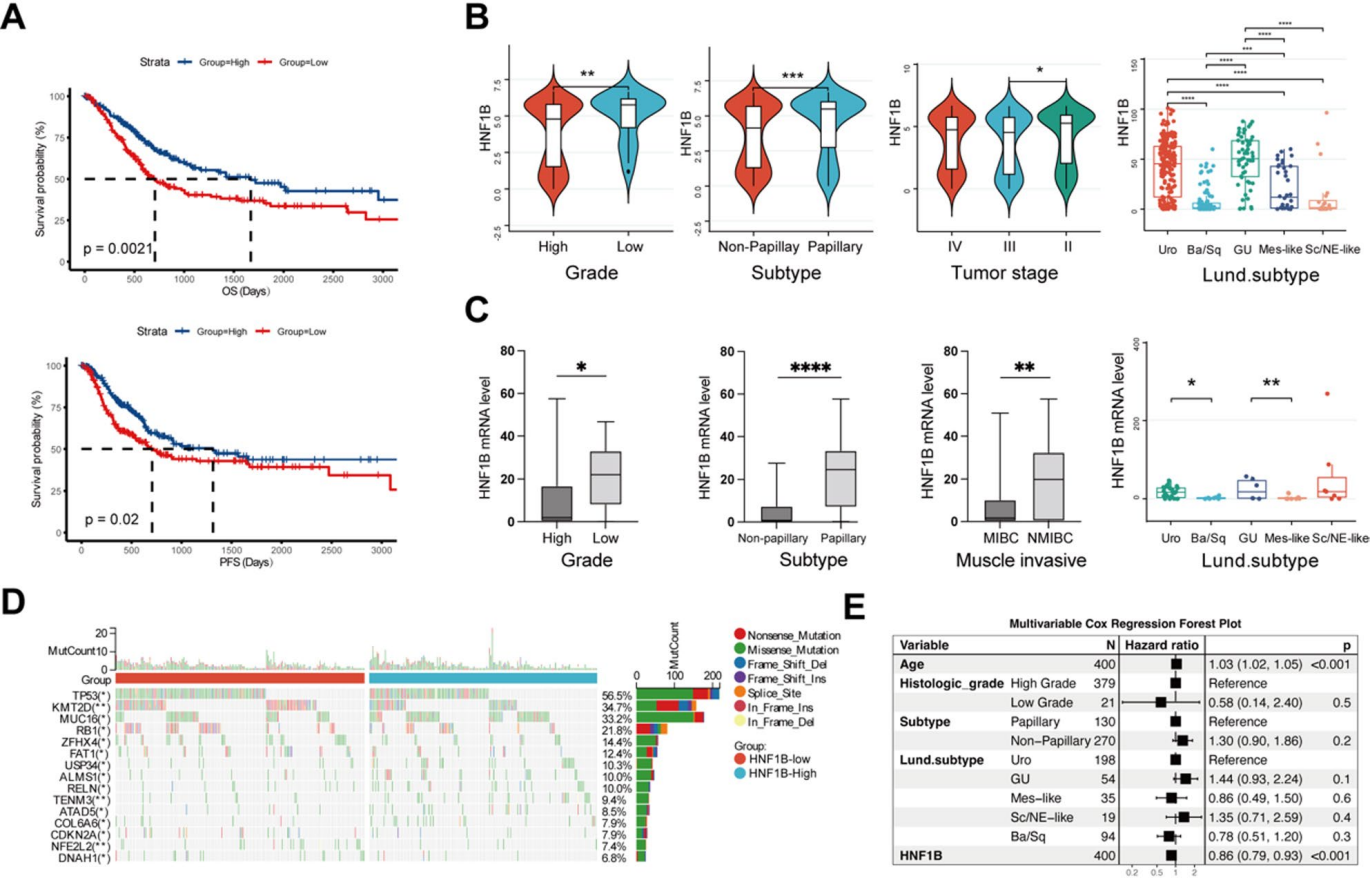
Association of HNF1B with prognosis and clinicopathological characteristics in BLCA. **A** Kaplan–Meier analysis of OS and PFS in the TCGA-BLCA cohort stratified by high vs. low HNF1B expression. **B** HNF1B expression levels across clinicopathological features and molecular subtypes (Urothelial-like [Uro], Genomically Unstable [GU], Basal/Squamous-like [Ba/Sq], Mesenchymal-like [Mes-like], Stem-like/Neuroendocrine-like [Sc/NE-like]) in the TCGA-BLCA cohort. **C** Validation of HNF1B expression associations with clinicopathological features (MIBC, muscle-invasive; NMIBC, non-muscle invasive) and molecular subtypes in the Xiangya cohort. **D** Mutational landscape of TCGA-BLCA tumors stratified by HNF1B expression (high vs. low). F. Forest plot from multivariable Cox regression analysis for OS in TCGA-BLCA cohort. *P < 0.05, **P < 0.01, ***P < 0.001, ****P < 0.0001

Analysis of TCGA-BLCA cohort identified low HNF1B expression as a marker of poor prognosis, associated with significantly shorter overall survival (OS) and progression-free survival (PFS) compared to high-expression patients (Fig. 1A). Furthermore, HNF1B expression was significantly lower in patients with aggressive clinicopathological features, including high grade, non-papillary, and locally advanced (Stage III) BLCA (Fig. 1B). HNF1B expression was lower in the more aggressive Lund subtypes: the Basal/Squamous (Ba/Sq), defined by its signature markers; the Mesenchymal-like (Mes-like), known for its invasive and stromal-rich phenotype; and the Stem-like/Neuroendocrine-like (Sc/NE-like), a rare but highly lethal variant (Fig. 1B). Analysis of the independent Xiangya cohort confirmed significantly reduced HNF1B expression in high-grade, non-papillary, and muscle-invasive BLCA samples (Fig. 1C). Regarding molecular subtypes, HNF1B expression was lower in the Ba/Sq and Mes-like subtypes compared to other molecular subtypes (Fig. 1C). Although no significant survival difference was observed in the Xiangya cohort, likely due to its limited size and follow-up (Fig. S1A). Analysis of TCGA-BLCA cohort revealed that HNF1B-low tumors exhibited a higher tumor mutational burden, with frequent mutations in pivotal genes such as TP53, KMT2D, and MUC16 (Fig. 1D). To evaluate the independent prognostic value of HNF1B, we performed multivariate Cox regression analysis on the TCGA-BLCA cohort, adjusting for clinicopathological variables. This analysis established HNF1B as a favorable prognostic factor in BLCA, independent of age, molecular subtype, and other key clinical features (Fig. 1E).

Analysis of the TCGA-BLCA cohort showed no significant difference in HNF1B mRNA levels between tumors and adjacent normal tissues (Fig. S1B), though this finding may require validation in larger cohorts given the limited number of normal samples. IHC images from the HPA database demonstrate predominant nuclear localization of HNF1B in normal urothelium. Furthermore, HPA data suggest that HNF1B expression may be reduced in a subset of BLCA cases (Fig. S1C).

### HNF1B inhibits the proliferation, migration, and invasion of BLCA cells

**Fig. 2:**
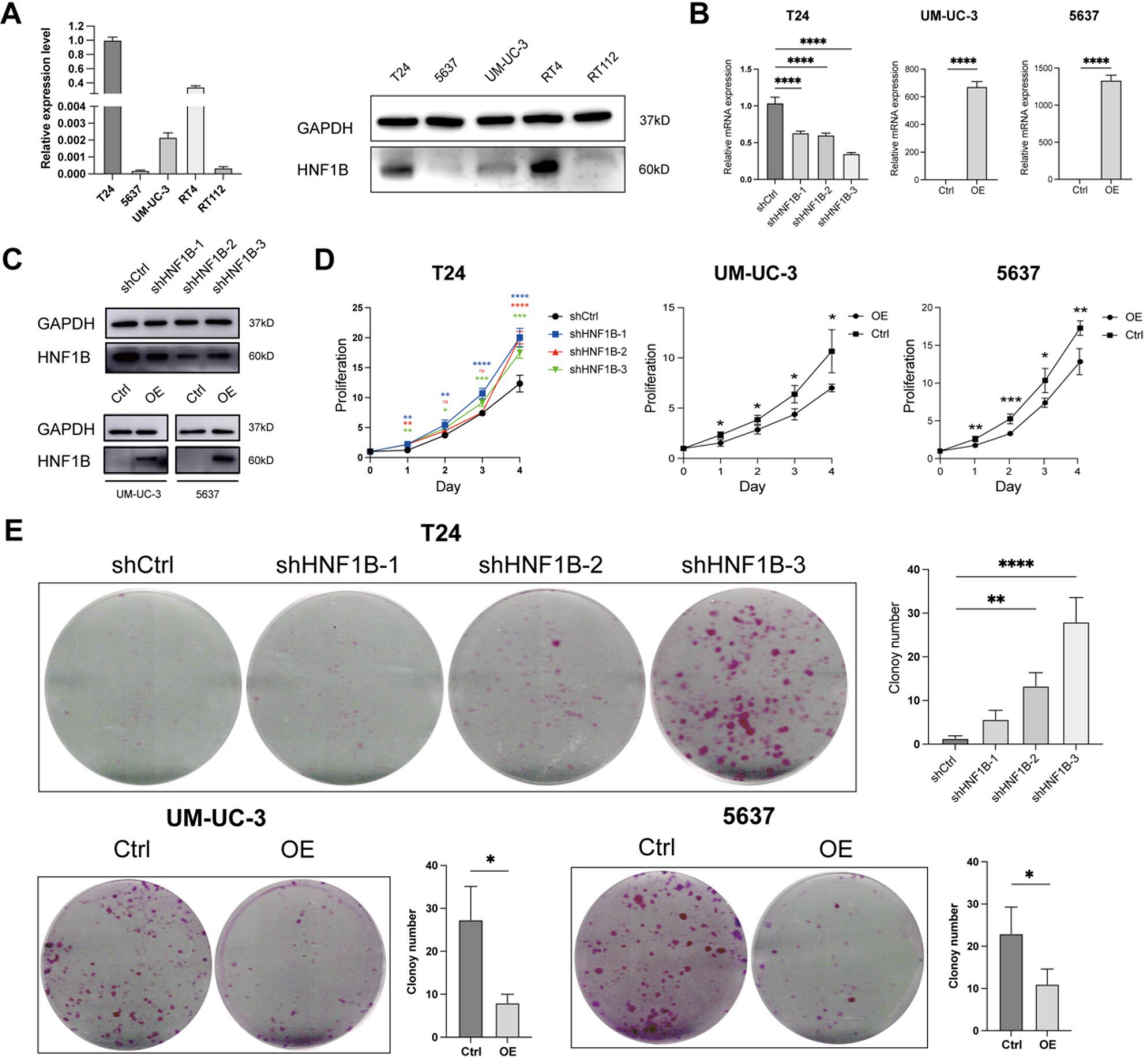
HNF1B expression and its functional impact on BLCA cell proliferation. **A** HNF1B expression at mRNA and protein levels across five BLCA cell lines. **B**, **C** Validation of HNF1B knockdown (shHNF1B-1/2/3 vs. shCtrl) and overexpression (OE vs. Ctrl) efficiency by qRT-PCR (**B**) and Western blot (**C**). **D** Cell proliferation measured by CCK-8 assay following HNF1B knockdown or overexpression. **E** Clonogenic survival assessed by colony formation assay following HNF1B knockdown or overexpression. ns, not significant, *P < 0.05, **P < 0.01, ***P < 0.001, ****P < 0.0001

We profiled HNF1B expression across five BLCA cell lines (Fig. 2A). To model HNF1B's role in aggressive BLCA, we prioritized cell lines with well-characterized invasive properties and relevance to disease progression. Therefore, we focused on T24 (for knockdown) and 5637/UM-UC-3 (for overexpression), which are established models for studying invasion, metastasis, and in vivo tumorigenesis (Fig. 2B, C).

Using the CCK-8 assay, we found that knockdown of HNF1B promoted T24 cell proliferation, whereas its overexpression suppressed proliferation in 5637/UM-UC-3 cells (Fig. 2D). Consistent with this, colony formation assays showed that HNF1B knockdown increased, while its overexpression markedly decreased, clonogenic capacity (Fig. 2E).

**Fig. 3:**
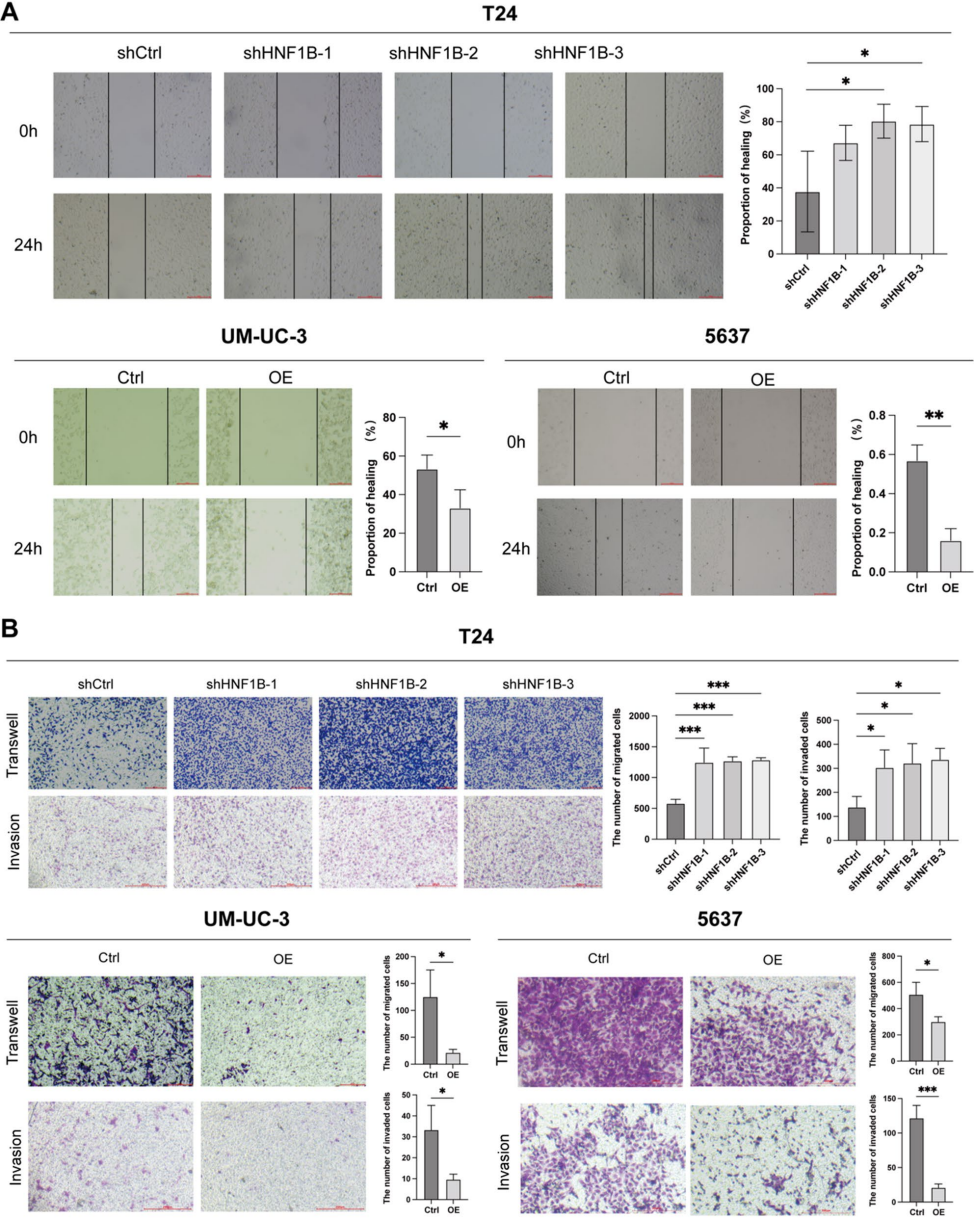
Regulatory role of HNF1B in BLCA cell migration and invasion. **A** Effects of HNF1B knockdown or overexpression on cell migration, assessed by wound healing assays. **B** Effects of HNF1B knockdown or overexpression on cell migration and invasion, assessed by Transwell assays. *P < 0.05, **P < 0.01, ***P < 0.001

In wound healing assays, HNF1B knockdown accelerated T24 cell migration, whereas its overexpression impeded migration in 5637 and UM-UC-3 cells (Fig. 3A). Consistent with this, Transwell assays showed that HNF1B knockdown promoted both migration and invasion of T24 cells, while its overexpression attenuated these processes in 5637 and UM-UC-3 cells (Fig. 3B).

To further substantiate these findings, we employed complementary approaches by overexpressing HNF1B in T24 cells and knocking it down in 5637 cells using lentiviral vectors (Fig. S2A). Consistent with its tumor-suppressive role, HNF1B overexpression in T24 cells suppressed proliferation and migration (Fig. S2B, D, E, and G), whereas its knockdown in 5637 cells moderately enhanced both phenotypes (Fig. S2B, C, E, and F), collectively supporting HNF1B as a negative regulator of malignant behaviors in BLCA.

### Overexpression of HNF1B inhibits BLCA tumor growth in vivo

**Fig. 4:**
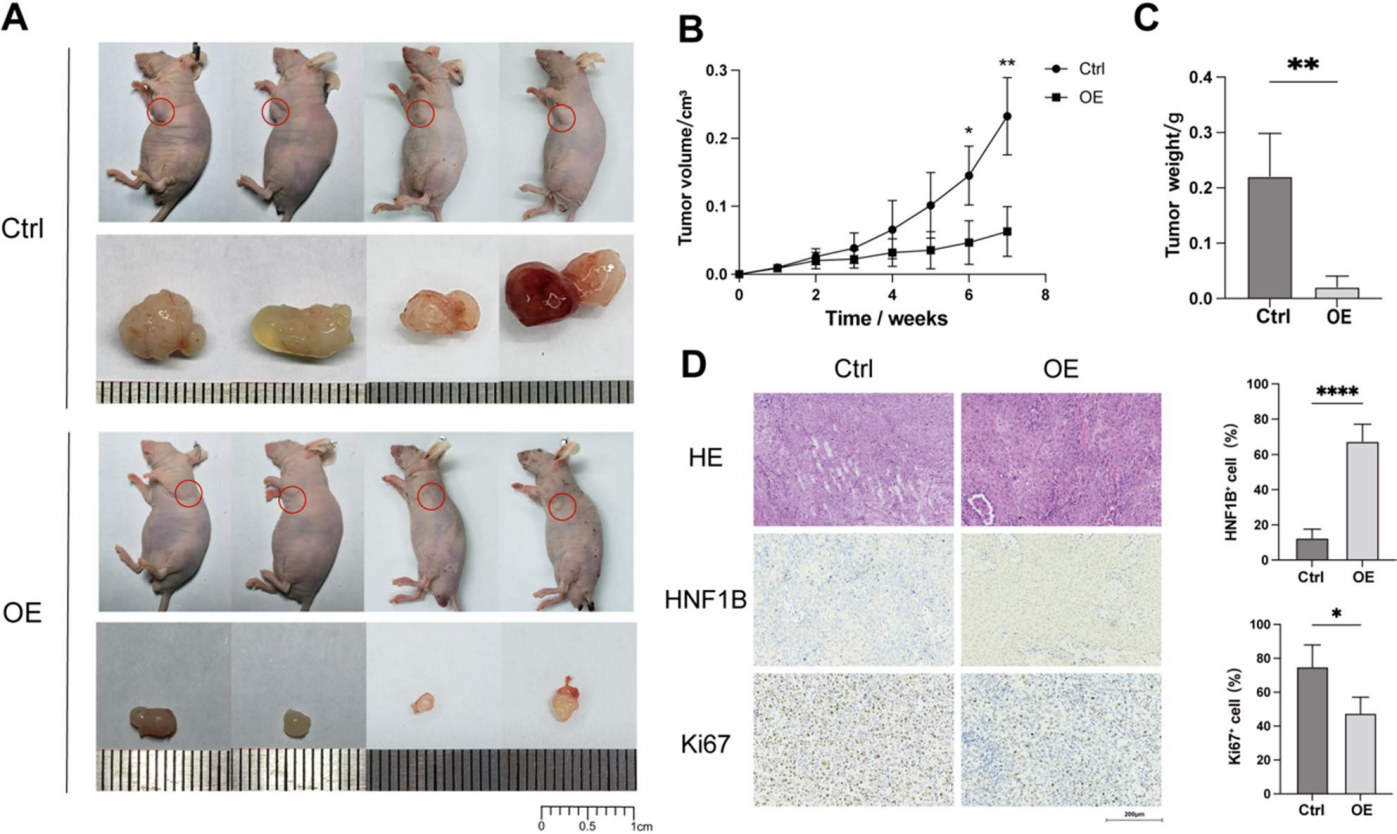
In vivo tumor-suppressive effect of HNF1B in BLCA. **A** Macroscopic appearance of mice and xenograft tumors at endpoint (OE vs. Ctrl). **B** Xenograft growth kinetics (OE vs. Ctrl). **C** Comparison of final xenograft weights (OE vs. Ctrl). **D** Representative HE and IHC (HNF1B, Ki67) staining in tumor sections from the indicated groups. * *P* < 0.05, ** *P* < 0.01, **** *P* < 0.0001

To validate the tumor-suppressive role of HNF1B in vivo, we established xenograft models by subcutaneously implanting 5637 cells stably overexpressing HNF1B or control vectors into nude mice. Notably, HNF1B overexpression markedly impaired tumor formation, with xenografts being difficult to establish. Among the tumors that did form, HNF1B overexpression significantly inhibited their growth compared to controls (Fig. 4A–C). IHC analysis confirmed higher HNF1B expression and a correspondingly lower Ki67-positive rate in the overexpression group (Fig. 4D), corroborating the suppressed proliferative activity in vivo.

### The decreased expression of HNF1B in BLCA is attributed to DNA hypermethylation

**Fig. 5:**
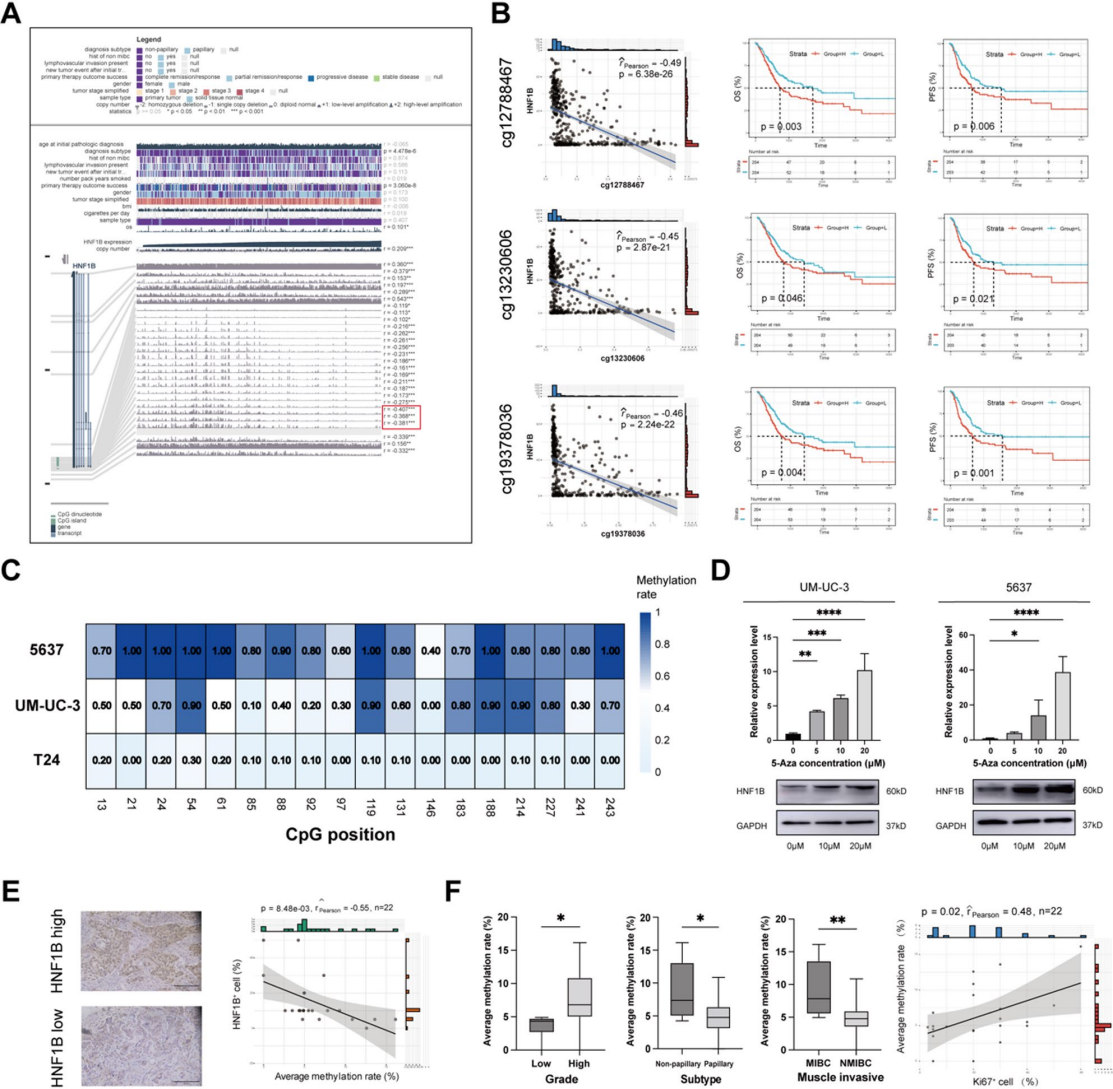
Epigenetic regulation of HNF1B via promoter methylation in BLCA. **A** Correlation between DNA methylation across HNF1B gene region and its mRNA levels in TCGA-BLCA cohort. **B** Association between methylation at HNF1B promoter sites (cg12788467, cg13230606, cg19378036) and patient prognosis in TCGA-BLCA cohort. **C** HNF1B promoter methylation patterns in three BLCA cell lines assessed by BSP. **D** Reactivation of HNF1B expression 5-AzaC treatment in UM-UC-3 and 5637 cells. **E** Correlation between HNF1B promoter methylation and expression in an independent clinical cohort. F. Association between HNF1B promoter methylation and clinicopathological variables in the clinical cohort. *P < 0.05, **P < 0.01, ***P < 0.001, ****P < 0.0001

To investigate the regulatory mechanism of HNF1B in BLCA, we performed an integrated genomic and epigenomic analysis using the GSCA platform (Fig. S3A-C). This revealed a significant inverse correlation was observed between HNF1B mRNA levels and promoter methylation at the cg19378036 site in the TCGA-BLCA cohort (Fig. S3A). Marked hypermethylation at this specific CpG site was identified in BLCA tissues compared to normal adjacent tissues from the same cohort, supporting the notion that DNA methylation-mediated silencing is a cancer-specific mechanism regulating HNF1B expression (Fig. S3D-E). Furthermore, we utilized TCGA-BLCA cohort to examine the correlation between expression and DNA methylation across its gene region. Methylation at multiple promoter CpG sites showed a significant, but moderate correlation with HNF1B expression (Fig. 5A). To identify clinically relevant sites, we selected three promoter CpG sites with the strongest negative correlation (cg12788467, cg13230606, cg19378036) for prognostic analysis. Hypermethylation at each site was significantly associated with shorter OS and PFS (Fig. 5B).

To validate promoter methylation as a mechanism for HNF1B silencing, we performed BSP in three BLCA cell lines. Consistent with their low endogenous HNF1B expression, 5637 and UM-UC-3 cells exhibited promoter hypermethylation (average rates: 55.6% and 83.9%, respectively). In stark contrast, the HNF1B-high T24 cells showed minimal promoter methylation (average rate: 8.9%) (Fig. 5C, Fig. S3F). Treatment with the DNA methyltransferase inhibitor 5-AzaC significantly restored HNF1B expression in 5637 and UM-UC-3 cells (Fig. 5D), confirming promoter hypermethylation as a key silencing mechanism. Extending these findings to clinical samples, pyrosequencing of 22 BLCA tissues revealed a significant negative correlation between average HNF1B promoter methylation rate and its expression (r =  − 0.55, *P* = 0.008; Fig. 5E, Table S6). In this pyrosequencing cohort, higher methylation rates were associated with aggressive clinicopathological features (high grade, non-papillary, muscle invasion) and positively correlated with the Ki67 positivity rate (r = 0.48, *P* = 0.02; Fig. 5F).

### HNF1B exerts an inhibitory effect on the BLCA progression by suppressing the MAPK pathway

**Fig. 6:**
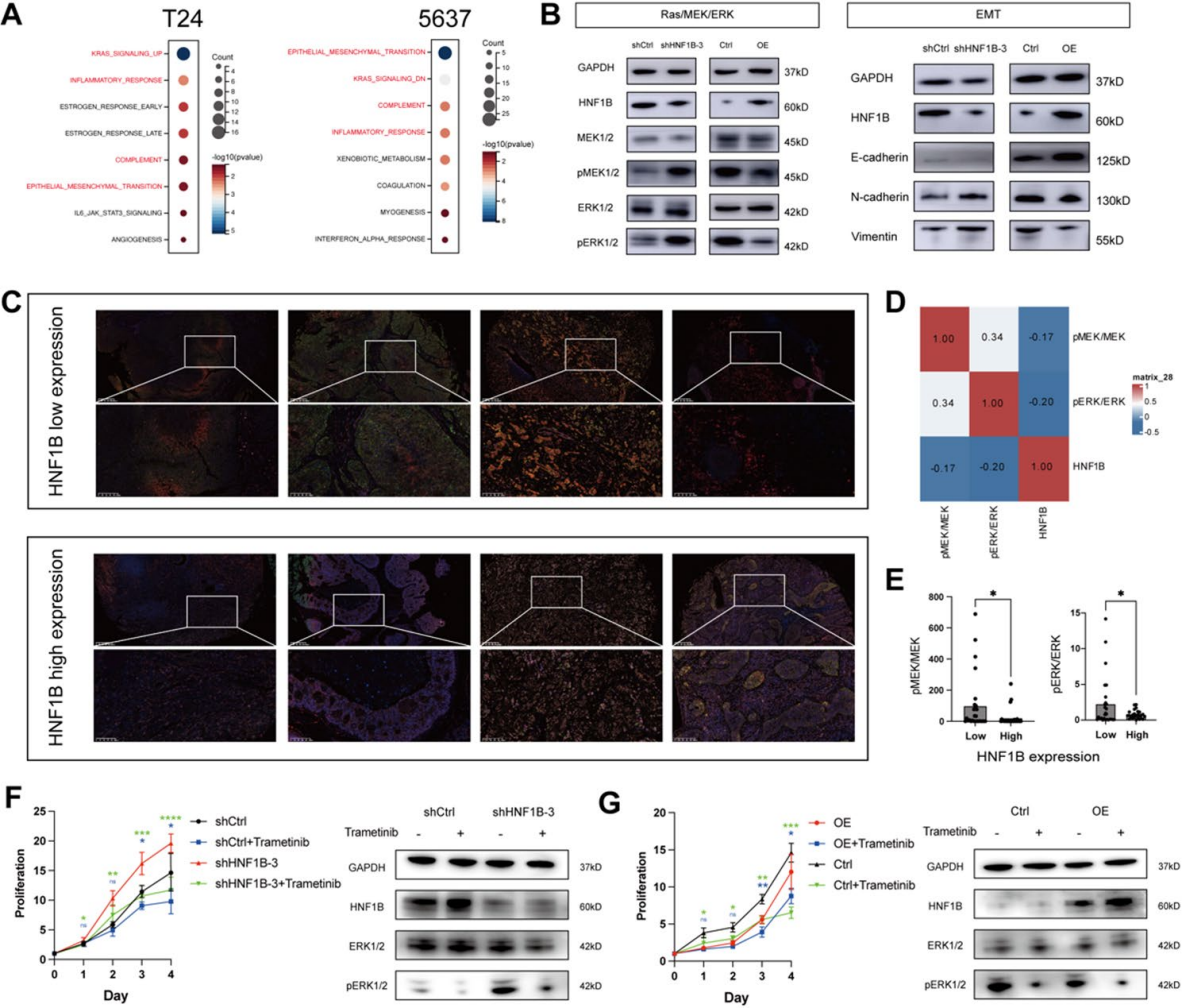
Downstream signaling and validation of HNF1B in BLCA. **A** Hallmark gene set enrichment analysis of DEGs from HNF1B-knockdown T24 (shHNF1B-3 vs. shCtrl) and HNF1B-overexpressing 5637 (OE vs. Ctrl) cells. **B** Protein expression analysis of MAPK/EMT pathways in HNF1B-knockdown T24 (shHNF1B-3 vs. shCtrl) and HNF1B-overexpressing 5637 (OE vs. Ctrl) cells. **C** Representative images of multiplex immunofluorescence analysis of HNF1B and MAPK pathway activity in clinical BLCA samples. Chromatic assignments: blue (DAPI), green (pMEK1/2), red (pERK1/2), pink (HNF1B), orange (MEK1/2), purple (ERK1/2). **D** Correlation heatmap of HNF1B expression and MAPK pathway activity. **E** Comparison of MAPK pathway activity grouped by HNF1B expression. **F**, **G** Pharmacological rescue with trametinib in HNF1B-knockdown T24 (**F**) and HNF1B-overexpressing 5637 (**G**) cells. Statistical comparisons were performed within each cell line between the vehicle and trametinib-treated groups, with significance denoted by asterisks above the corresponding colored bars/lines. ns, not significant, *P < 0.05, **P < 0.01, ***P < 0.001

To investigate the mechanism underlying the anti-tumor activity of HNF1B in BLCA, we performed RNA sequencing on HNF1B-knockdown T24 and HNF1B-overexpressing 5637 cells. This identified 975 DEGs between the control and HNF1B knockdown groups (492 up/483 down; Table S7) and 753 DEGs between the control and HNF1B overexpression groups (299 up/454 down; Table S8). Enrichment analysis of Hallmark gene sets demonstrated that the DEGs from both HNF1B knockdown and overexpression experiments were consistently enriched in four key pathways: KRAS signaling, inflammatory response, complement, and EMT (Fig. 6A).

To identify direct targets of the transcription factor HNF1B mediating its pathway effects, we integrated DEGs from T24 and 5637 cells, identifying, identifying 26 candidate genes (8 down, 18 up; Fig. S4A). Among these, correlation analysis in the TCGA-BLCA cohort revealed 9 genes whose expression was consistently and significantly correlated with HNF1B, including 4 negatively correlated genes (FGFBP1, CXCL10, CXCL11, RSAD2) and 5 positively correlated genes (PPFIBP2, PRRT3, CABP1, EXTL1, SLC29A4) (Fig. S4B).

To investigate the relationship between HNF1B and KRAS signaling, we first analyzed the association between HNF1B expression and mutations in core KRAS pathway genes (including KRAS, NRAS, HRAS, and BRAF) in TCGA-BLCA cohort, but found no significant correlation (Fig. S4C). Then, we focused on its critical downstream cascade, the MAPK pathway. Cell-based experiments demonstrated that HNF1B knockdown increased phosphorylation of MEK1/2 (Ser218/222) and ERK1/2 (Thr202/Tyr204), while its overexpression decreased their phosphorylation, without affecting their total protein levels (Fig. 6B), indicating that HNF1B negatively regulates MAPK pathway activity. As for EMT, our investigation revealed that HNF1B knockdown increased levels of the mesenchymal markers N-cadherin and vimentin. Conversely, its overexpression decreased these markers and elevated the epithelial marker E-cadherin (Fig. 6B). These findings indicated that HNF1B suppresses the EMT in BLCA.

To clinically validate HNF1B's inhibition on the MAPK pathway, we performed multiplex immunofluorescence staining on a BLCA tissue microarray (53 evaluable samples; Fig. 6C, Table S9), assessing the correlation between HNF1B expression and MAPK activity (pMEK/MEK, pERK/ERK ratios). Although a non-significant inverse trend was observed (all *P* > 0.05, Fig. 6D), dichotomized analysis (median cutoff) revealed that the HNF1B-low group had significantly elevated pMEK/MEK and pERK/ERK ratios compared to the HNF1B-high group (Fig. 6E), supporting HNF1B as a MAPK pathway inhibitor in BLCA tissues. We also performed rescue experiments to validate the mechanism. HNF1B-knockdown/control T24 cells and HNF1B-overexpressing/control 5637 cells were treated with optimized concentrations of the MEK inhibitor trametinib (10 μM and 5 μM, respectively; Fig. S5A). The results demonstrated that trametinib significantly suppressed the hyperproliferative phenotype induced by low HNF1B expression (Fig. 6F, G). At the molecular level, Western blot analysis confirmed trametinib's inhibitory effect on the MAPK pathway, as evidenced by markedly reduced ERK1/2 phosphorylation (Fig. 6F, G). These results support the conclusion that HNF1B modulates cell proliferation, at least in part, through its regulatory influence on the MAPK pathway.

Furthermore, we employed transcriptome sequencing to investigate the effects of HNF1B on cell cycle and apoptosis. GSEA analysis of the Hallmark gene sets revealed that in HNF1B-high-expressing cell lines exhibited significant downregulation of cell cycle-related pathways (*e.g.*, E2F Targets and G2M Checkpoint) and upregulation of the apoptosis-related p53 pathway. Consistent results were obtained from GSEA analysis of KEGG pathways, which showed suppression of the cell cycle pathway and enhancement of apoptosis-related pathways (including Apoptosis and p53 signaling) in these cells (Fig. S5B-C). These findings suggest that the tumor-suppressive role of HNF1B may be mediated through inhibition of cell cycle progression and promotion of apoptotic processes.

## Discussion

The epigenetic silencing of tumor suppressor genes is a key driver of cancer progression. Here, we demonstrate that the transcription factor HNF1B is a frequent target of this mechanism in BLCA. Specifically, promoter DNA methylation leads to transcriptional repression of HNF1B. Functional studies established that HNF1B loss promotes tumor aggressiveness by enhancing cell proliferation and invasion. These findings position HNF1B as a critical epigenetically regulated tumor suppressor in BLCA and suggest its promoter methylation status as a potential biomarker for disease stratification.

Analyses of public datasets and the Xiangya cohort consistently demonstrated that low HNF1B expression correlates with poor prognosis and aggressive features in BLCA, suggesting that HNF1B serves as a protective factor against BLCA. However, these data remained associative. To move beyond correlation and define its functional role, we performed gain- and loss-of-function studies. Both in vitro and in vivo experiments demonstrated that HNF1B acts as a tumor suppressor, directly inhibiting BLCA cell proliferation and migration.

To explore the upstream mechanisms underlying HNF1B downregulation in BLCA, we focused on epigenetic regulation. The epigenetic landscape, particularly DNA methylation, has emerged as a fundamental regulator of oncogene and tumor suppressor activity in cancer [20]. Mechanistically, we identified DNA methylation as a key mechanism silencing HNF1B expression in BLCA. The methylation-mediated silencing of HNF1B observed in BLCA mirrors established patterns in other malignancies. HNF1B promoter hypermethylation is a recurrent epigenetic event across multiple malignancies. For instance, it has been correlated with adverse clinicopathological features in ovarian cancer [21], higher Gleason score and advanced stage in prostate cancer [22], tumor recurrence in colorectal carcinoma [23], and poor differentiation in lung adenocarcinoma [21, 22, 24, 25]. Bioinformatic analysis of the TCGA-BLCA cohort identified a significant negative correlation between HNF1B expression and promoter DNA methylation. BSP confirmed HNF1B promoter hypermethylation in low-expressing BLCA cells, and treatment with the DNA methyltransferase inhibitor 5-AzaC restored HNF1B expression. Moreover, pyrosequencing of 22 primary BLCA tissues independently confirmed the inverse correlation between promoter methylation and HNF1B expression. Collectively, our study integrates multi-level evidence from public cohort, cellular models, and clinical samples to establish DNA methylation as a principal mechanism for silencing HNF1B in BLCA. To translate these findings into clinical practice, future studies should evaluate the prognostic utility of HNF1B promoter methylation and its potential as a biomarker for guiding epigenetic therapies in BLCA. In addition, we noticed a correlation between HNF1B expression and CNV. Systematic investigation of the genomic landscape surrounding HNF1B will be valuable to elucidate the combinatorial role of genetic and epigenetic alterations in BLCA progression.

Emerging evidence has established HNF1B as a pivotal regulator of oncogenic pathways across multiple cancer types, including pancreatic and prostate cancers. Our study extends these findings to BLCA, in which we identified the MAPK pathway and EMT as key mechanisms through which HNF1B influences tumor progression. A study in pancreatic cancer have shown that HNF1B deficiency cooperates with oncogenic KRAS to promote pancreatic intraepithelial neoplasia initiation and progression [26]. Mechanistically, HNF1B exerts this effect, at least in part, through direct transcriptional regulation of PTF1A. The loss of PTF1A in turn sensitizes acinar cells to KRAS-driven transformation [27]. These findings collectively position HNF1B, in pancreatic cancer, as an upstream modulator of cellular sensitivity to oncogenic KRAS signaling. In prostate cancer, HNF1B has been shown to inhibit EMT through downregulation of SLUG [13]. Although our findings in BLCA suggest that HNF1B modulates MAPK pathway and EMT process, the precise underlying mechanisms warrant further investigation. To further elucidate the underlying mechanisms, we identified nine candidate HNF1B target genes in BLCA. Notably, FGFBP1, which is significantly upregulated upon HNF1B loss, functions as a molecular chaperone that mobilizes matrix-anchored fibroblast growth factors (FGFs), facilitating their release and subsequent binding to their receptors (FGFRs) to enhance signaling [28]. As a key upstream activator of the MAPK pathway, enhanced FGF signaling can stimulate MAPK cascade [29]. Thus, FGFBP1 may represent a crucial molecular bridge linking HNF1B loss to the activation of MAPK pathway. The pro-tumorigenic environment resulting from HNF1B loss may be further amplified by the dysregulation of chemokine signaling. In our study, we observed HNF1B loss was associated with an upregulation of CXCL10 and CXCL11 in BLCA. Given that both chemokines act through the CXCR3 receptor to induce an EMT-like phenotype and promote cell invasion in pancreatic cancer [30], this suggests a potential mechanism that could be operational in HNF1B-deficient BLCA. Furthermore, PPFIBP2 has been identified as a key suppressor of EMT in head and neck squamous cell carcinoma [31]. We propose that its upregulation by HNF1B constitutes a potential mechanism for EMT inhibition in BLCA.

The MAPK pathway is a validated therapeutic target in BLCA, with MEK inhibitors showing preclinical and clinical efficacy [32–35]. In this study, we observed an association between low HNF1B expression and elevated MAPK pathway activity in BLCA. In vitro, the MEK inhibitor trametinib potently suppressed hyperproliferation in HNF1B-deficient cells. This pattern merits further investigation to determine whether HNF1B expression could predict MAPK dependency and thus inform patient stratification strategies.

While our study provides important insights into the epigenetic regulation of HNF1B in BLCA, several limitations should be acknowledged. First, the sample size of our pyrosequencing cohort was limited, which may affect the statistical robustness and generalizability of the methylation findings. Furthermore, due to the lack of matched normal adjacent tissues in this cohort, we could not definitively establish whether the observed promoter hypermethylation of HNF1B is a tumor-specific epigenetic alteration in BLCA. Future studies incorporating larger sample sets and paired normal-tumor tissue comparisons are necessary to validate the reliability of these results and to confirm the cancer-specific nature of this regulatory mechanism. Second, while our transcriptomic analysis identified candidate genes linking HNF1B to MAPK/EMT regulation, their roles as direct transcriptional targets of HNF1B require comprehensive validation through combined approaches, including chromatin immunoprecipitation (ChIP), luciferase reporter assays, and functional rescue experiments. Additionally, the limited tumor formation necessitates cohort expansion and the inclusion of additional representative BLCA cell lines in future work. Notwithstanding these limitations, our study establishes a foundational framework for elucidating HNF1B's tumor-suppressive mechanisms and evaluating its therapeutic potential in BLCA.

## Conclusion

This study establishes DNA methylation as the key mechanism silencing the tumor suppressor HNF1B in BLCA, thereby activating the MAPK pathway and EMT to drive tumor progression. These findings define a targetable epigenetic vulnerability, suggesting that patients with HNF1B-low tumors may benefit from therapies designed to reverse this silencing or inhibit the activated downstream pathways.

## Supplementary Information

Below is the link to the electronic supplementary material.

Supplementary Fig. 1. Supplementary analysis of HNF1B in BLCA. **A**. Kaplan–Meier analysis of OS in the Xiangya cohort stratified by high vs. low HNF1B expression. **B**. Comparison of HNF1B mRNA levels between tumor and adjacent normal tissues in the TCGA-BLCA cohort. **C**. Representative IHC images of HNF1B in BLCA and normal urothelial tissues from the HPA. ns, not significant. Supplementary Fig. 2. Control experiments for HNF1B function in BLCA cells. **A**. Validation of HNF1B knockdown (shHNF1B-3 vs. shCtrl) and overexpression (OE vs. Ctrl) efficiency by qRT-PCR and Western blot. **B**. Cell proliferation measured by CCK-8 assay following HNF1B knockdown or overexpression. **C**, **D**. Clonogenic survival assessed by colony formation assay following HNF1B knockdown in 5637 cells (**C**) or overexpression in T24 cells (**D**). **E**. Effects of HNF1B knockdown or overexpression on cell migration, assessed by wound healing assays. **F**, **G**. Effects of HNF1B knockdown in 5637 cells (**F**) or overexpression in T24 cells (**G**) on cell migration and invasion, assessed by Transwell assays. ns, not significant, * P < 0.05, ** P < 0.01, *** P < 0.001, **** P < 0.0001. Supplementary Fig. 3. Comprehensive analysis of HNF1B genetic and epigenetic alterations in BLCA. **A**. Correlation between HNF1B expression and its promoter methylation at site cg19378036 in the TCGA-BLCA cohort. **B**. Analysis of HNF1B single nucleotide polymorphisms in the TCGA-BLCA cohort. **C**. Correlation between HNF1B expression and its CNV in the TCGA-BLCA cohort. **D**. DNA methylation across all HNF1B CpG sites in tumor (Ca) vs. normal (Nor) tissues in the TCGA-BLCA cohort **E**. Comparison of DNA methylation at three specific HNF1B promoter CpG sites in tumor vs. normal tissues in the TCGA-BLCA cohort. **F**. HNF1B promoter methylation in three BLCA cell lines assessed by BSP. ns, not significant, * P < 0.05. Supplementary Fig. 4. Integrated analysis of transcriptomic alterations and genetic landscape associated with HNF1B in BLCA. **A**. Comprehensive analysis of DEGs identified from RNA sequencing of HNF1B-knockdown T24 and HNF1B-overexpressing 5637 cells. **B**. Correlation analysis between HNF1B mRNA levels and those of its candidate target genes in the TCGA-BLCA cohort. **C**. Mutational landscape of core KRAS pathway genes stratified by HNF1B expression level (high vs. low) in the TCGA-BLCA cohort. Supplementary Fig. 5. Pharmacokinetic profiling of trametinib and transcriptomic GSEA in BLCA cells. **A**. Cell viability dose–response curves following 48-h gradient trametinib treatment in indicated BLCA cell lines. **B**, **C**. GSEA of cell cycle and apoptosis pathways in in HNF1B-knockdown T24 (**B**) and HNF1B-overexpressing 5637 (**C**) cells

## Data Availability

The data and materials in the current study are available from the corresponding author on reasonable request.
