# Supplementary figures and images for: DNA methylation-mediated silencing of HNF1B promotes bladder cancer progression
Source: Clin Epigenetics. 2026 Feb 21;18:91. doi: 10.1186/s13148-026-02079-z (PMC13202989; doi:10.1186/s13148-026-02079-z)

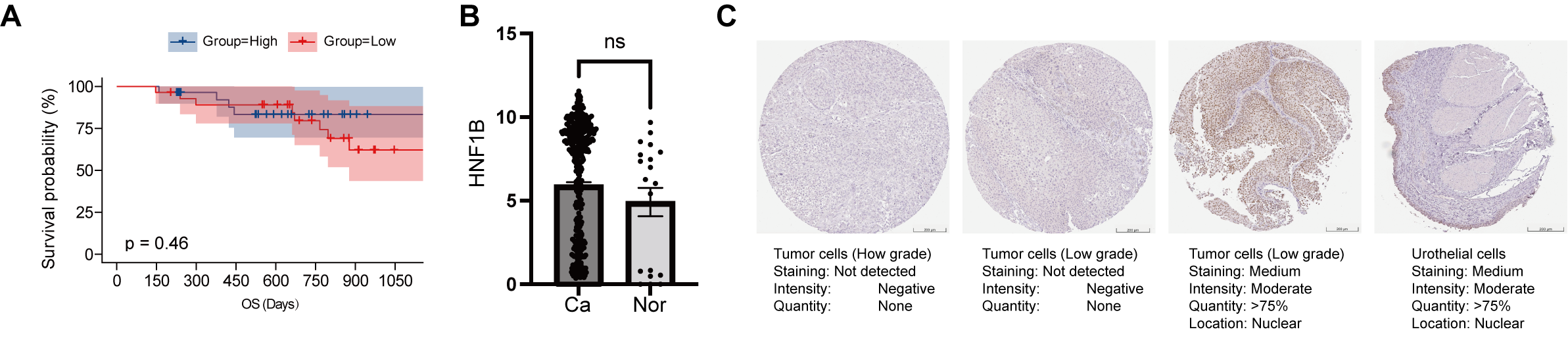

Supplement: Supplementary file 1 — Supplementary Fig. 1. Supplementary analysis of HNF1B in BLCA. A. Kaplan–Meier analysis of OS in the Xiangya cohort stratified by high vs. low HNF1B expression. B. Comparison of HNF1B mRNA levels between tumor and adjacent normal tissues in the TCGA-BLCA cohort. C. Representative IHC images of HNF1B in BLCA and normal urothelial tissues from the HPA. ns, not significant. Supplementary Fig. 2. Control experiments for HNF1B function in BLCA cells. A. Validation of HNF1B knockdown (shHNF1B-3 vs. shCtrl) and overexpression (OE vs. Ctrl) efficiency by qRT-PCR and Western blot. B. Cell proliferation measured by CCK-8 assay following HNF1B knockdown or overexpression. C, D. Clonogenic survival assessed by colony formation assay following HNF1B knockdown in 5637 cells (C) or overexpression in T24 cells (D). E. Effects of HNF1B knockdown or overexpression on cell migration, assessed by wound healing assays. F, G. Effects of HNF1B knockdown in 5637 cells (F) or overexpression in T24 cells (G) on cell migration and invasion, assessed by Transwell assays. ns, not significant, * P < 0.05, ** P < 0.01, *** P < 0.001, **** P < 0.0001. Supplementary Fig. 3. Comprehensive analysis of HNF1B genetic and epigenetic alterations in BLCA. A. Correlation between HNF1B expression and its promoter methylation at site cg19378036 in the TCGA-BLCA cohort. B. Analysis of HNF1B single nucleotide polymorphisms in the TCGA-BLCA cohort. C. Correlation between HNF1B expression and its CNV in the TCGA-BLCA cohort. D. DNA methylation across all HNF1B CpG sites in tumor (Ca) vs. normal (Nor) tissues in the TCGA-BLCA cohort E. Comparison of DNA methylation at three specific HNF1B promoter CpG sites in tumor vs. normal tissues in the TCGA-BLCA cohort. F. HNF1B promoter methylation in three BLCA cell lines assessed by BSP. ns, not significant, * P < 0.05. Supplementary Fig. 4. Integrated analysis of transcriptomic alterations and genetic landscape associated with HNF1B in BLCA. A. Comprehensive analysis of [file 13148_2026_2079_MOESM1_ESM.zip › Supplementary files/figS1.tif]

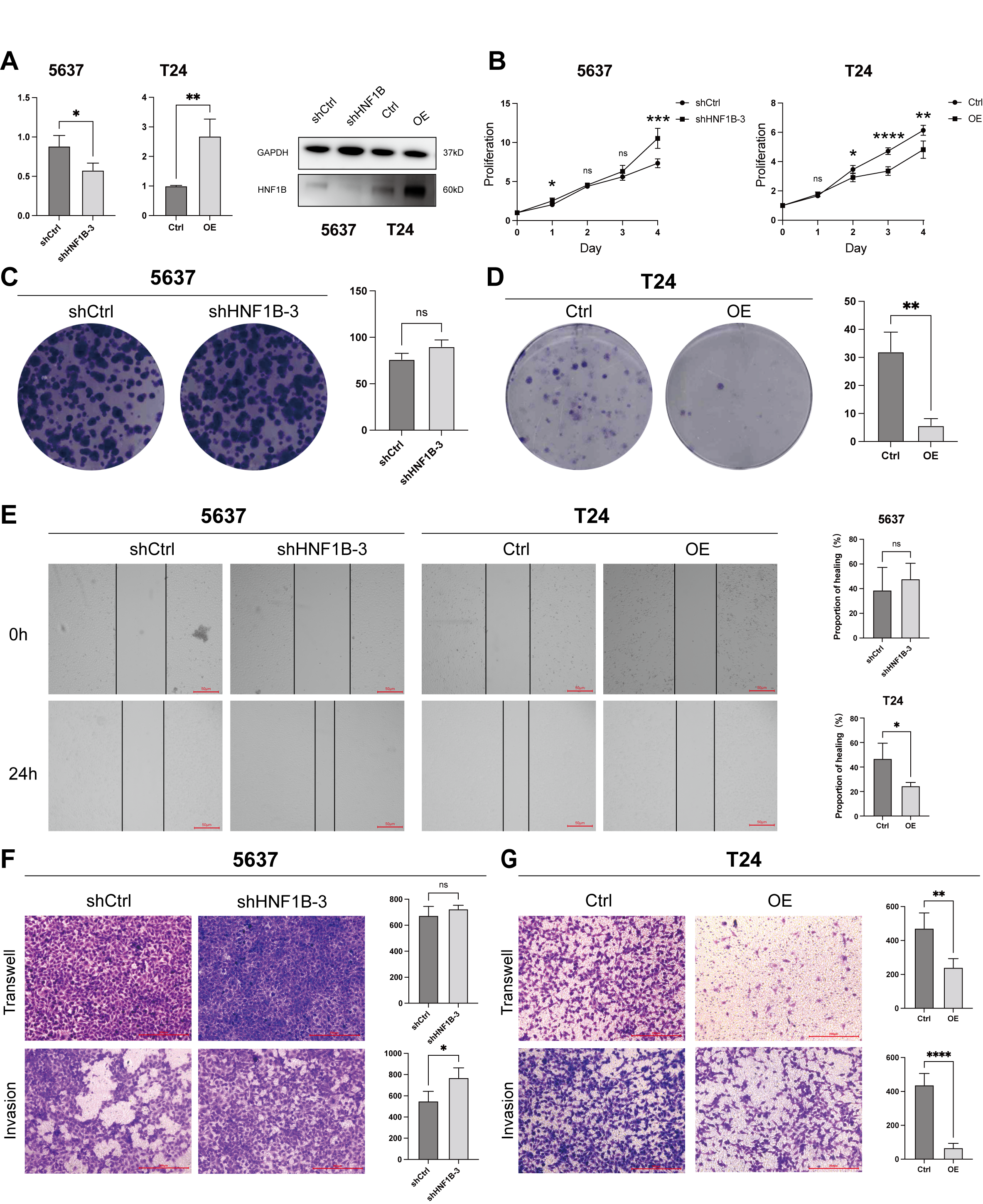

Supplement: Supplementary file 1 — Supplementary Fig. 1. Supplementary analysis of HNF1B in BLCA. A. Kaplan–Meier analysis of OS in the Xiangya cohort stratified by high vs. low HNF1B expression. B. Comparison of HNF1B mRNA levels between tumor and adjacent normal tissues in the TCGA-BLCA cohort. C. Representative IHC images of HNF1B in BLCA and normal urothelial tissues from the HPA. ns, not significant. Supplementary Fig. 2. Control experiments for HNF1B function in BLCA cells. A. Validation of HNF1B knockdown (shHNF1B-3 vs. shCtrl) and overexpression (OE vs. Ctrl) efficiency by qRT-PCR and Western blot. B. Cell proliferation measured by CCK-8 assay following HNF1B knockdown or overexpression. C, D. Clonogenic survival assessed by colony formation assay following HNF1B knockdown in 5637 cells (C) or overexpression in T24 cells (D). E. Effects of HNF1B knockdown or overexpression on cell migration, assessed by wound healing assays. F, G. Effects of HNF1B knockdown in 5637 cells (F) or overexpression in T24 cells (G) on cell migration and invasion, assessed by Transwell assays. ns, not significant, * P < 0.05, ** P < 0.01, *** P < 0.001, **** P < 0.0001. Supplementary Fig. 3. Comprehensive analysis of HNF1B genetic and epigenetic alterations in BLCA. A. Correlation between HNF1B expression and its promoter methylation at site cg19378036 in the TCGA-BLCA cohort. B. Analysis of HNF1B single nucleotide polymorphisms in the TCGA-BLCA cohort. C. Correlation between HNF1B expression and its CNV in the TCGA-BLCA cohort. D. DNA methylation across all HNF1B CpG sites in tumor (Ca) vs. normal (Nor) tissues in the TCGA-BLCA cohort E. Comparison of DNA methylation at three specific HNF1B promoter CpG sites in tumor vs. normal tissues in the TCGA-BLCA cohort. F. HNF1B promoter methylation in three BLCA cell lines assessed by BSP. ns, not significant, * P < 0.05. Supplementary Fig. 4. Integrated analysis of transcriptomic alterations and genetic landscape associated with HNF1B in BLCA. A. Comprehensive analysis of [file 13148_2026_2079_MOESM1_ESM.zip › Supplementary files/figS2.tif]

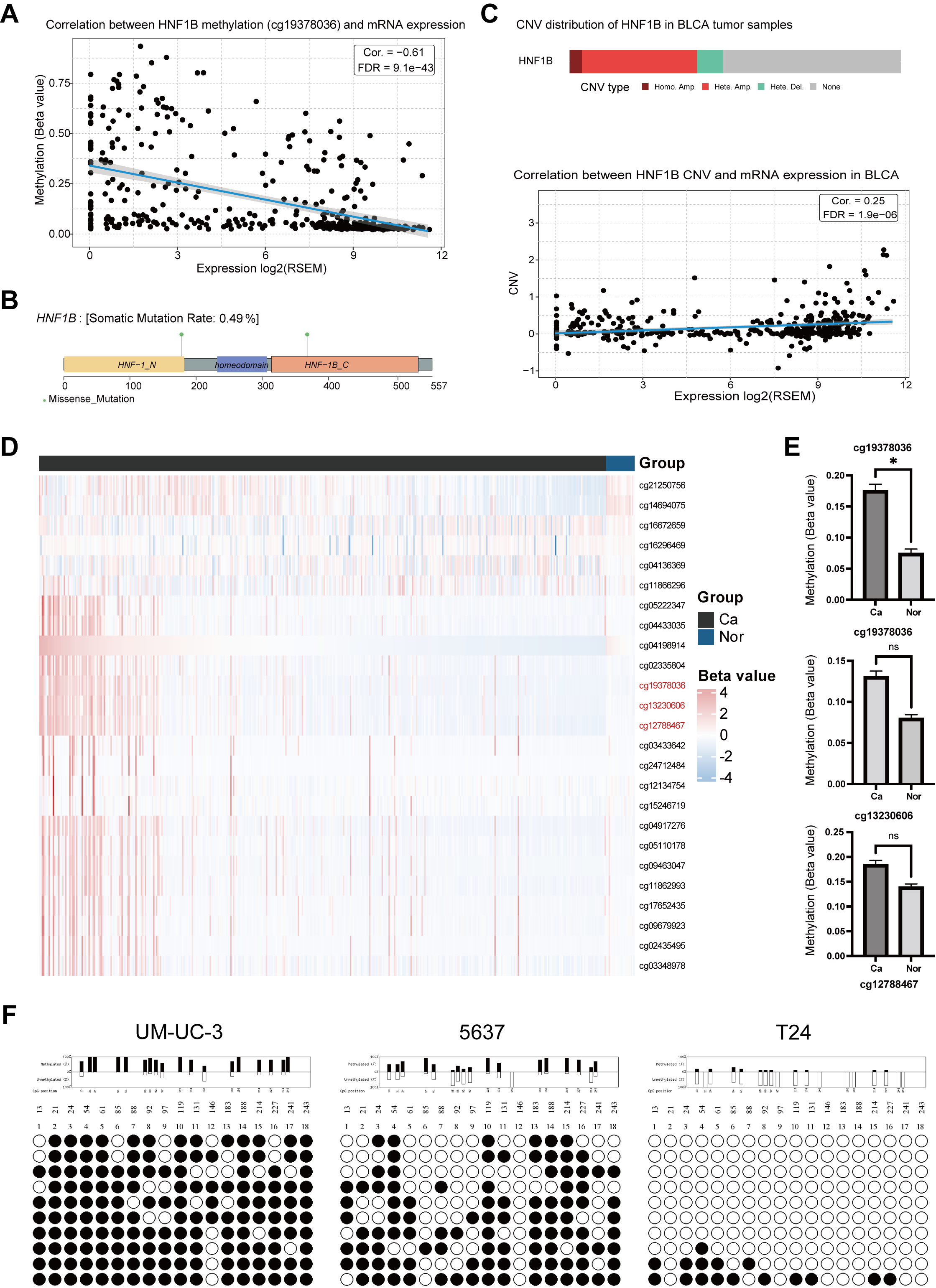

Supplement: Supplementary file 1 — Supplementary Fig. 1. Supplementary analysis of HNF1B in BLCA. A. Kaplan–Meier analysis of OS in the Xiangya cohort stratified by high vs. low HNF1B expression. B. Comparison of HNF1B mRNA levels between tumor and adjacent normal tissues in the TCGA-BLCA cohort. C. Representative IHC images of HNF1B in BLCA and normal urothelial tissues from the HPA. ns, not significant. Supplementary Fig. 2. Control experiments for HNF1B function in BLCA cells. A. Validation of HNF1B knockdown (shHNF1B-3 vs. shCtrl) and overexpression (OE vs. Ctrl) efficiency by qRT-PCR and Western blot. B. Cell proliferation measured by CCK-8 assay following HNF1B knockdown or overexpression. C, D. Clonogenic survival assessed by colony formation assay following HNF1B knockdown in 5637 cells (C) or overexpression in T24 cells (D). E. Effects of HNF1B knockdown or overexpression on cell migration, assessed by wound healing assays. F, G. Effects of HNF1B knockdown in 5637 cells (F) or overexpression in T24 cells (G) on cell migration and invasion, assessed by Transwell assays. ns, not significant, * P < 0.05, ** P < 0.01, *** P < 0.001, **** P < 0.0001. Supplementary Fig. 3. Comprehensive analysis of HNF1B genetic and epigenetic alterations in BLCA. A. Correlation between HNF1B expression and its promoter methylation at site cg19378036 in the TCGA-BLCA cohort. B. Analysis of HNF1B single nucleotide polymorphisms in the TCGA-BLCA cohort. C. Correlation between HNF1B expression and its CNV in the TCGA-BLCA cohort. D. DNA methylation across all HNF1B CpG sites in tumor (Ca) vs. normal (Nor) tissues in the TCGA-BLCA cohort E. Comparison of DNA methylation at three specific HNF1B promoter CpG sites in tumor vs. normal tissues in the TCGA-BLCA cohort. F. HNF1B promoter methylation in three BLCA cell lines assessed by BSP. ns, not significant, * P < 0.05. Supplementary Fig. 4. Integrated analysis of transcriptomic alterations and genetic landscape associated with HNF1B in BLCA. A. Comprehensive analysis of [file 13148_2026_2079_MOESM1_ESM.zip › Supplementary files/figS3.tif]

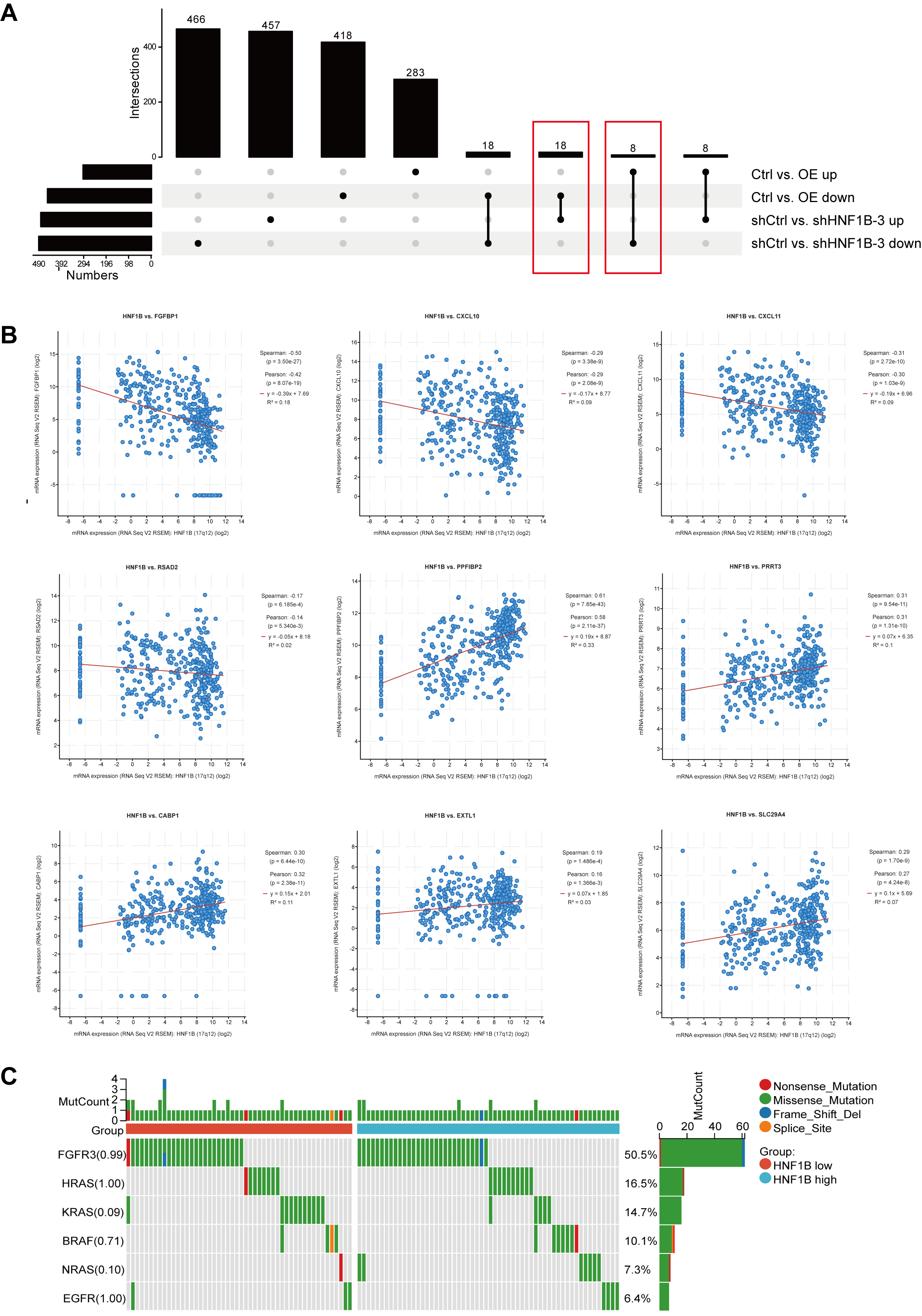

Supplement: Supplementary file 1 — Supplementary Fig. 1. Supplementary analysis of HNF1B in BLCA. A. Kaplan–Meier analysis of OS in the Xiangya cohort stratified by high vs. low HNF1B expression. B. Comparison of HNF1B mRNA levels between tumor and adjacent normal tissues in the TCGA-BLCA cohort. C. Representative IHC images of HNF1B in BLCA and normal urothelial tissues from the HPA. ns, not significant. Supplementary Fig. 2. Control experiments for HNF1B function in BLCA cells. A. Validation of HNF1B knockdown (shHNF1B-3 vs. shCtrl) and overexpression (OE vs. Ctrl) efficiency by qRT-PCR and Western blot. B. Cell proliferation measured by CCK-8 assay following HNF1B knockdown or overexpression. C, D. Clonogenic survival assessed by colony formation assay following HNF1B knockdown in 5637 cells (C) or overexpression in T24 cells (D). E. Effects of HNF1B knockdown or overexpression on cell migration, assessed by wound healing assays. F, G. Effects of HNF1B knockdown in 5637 cells (F) or overexpression in T24 cells (G) on cell migration and invasion, assessed by Transwell assays. ns, not significant, * P < 0.05, ** P < 0.01, *** P < 0.001, **** P < 0.0001. Supplementary Fig. 3. Comprehensive analysis of HNF1B genetic and epigenetic alterations in BLCA. A. Correlation between HNF1B expression and its promoter methylation at site cg19378036 in the TCGA-BLCA cohort. B. Analysis of HNF1B single nucleotide polymorphisms in the TCGA-BLCA cohort. C. Correlation between HNF1B expression and its CNV in the TCGA-BLCA cohort. D. DNA methylation across all HNF1B CpG sites in tumor (Ca) vs. normal (Nor) tissues in the TCGA-BLCA cohort E. Comparison of DNA methylation at three specific HNF1B promoter CpG sites in tumor vs. normal tissues in the TCGA-BLCA cohort. F. HNF1B promoter methylation in three BLCA cell lines assessed by BSP. ns, not significant, * P < 0.05. Supplementary Fig. 4. Integrated analysis of transcriptomic alterations and genetic landscape associated with HNF1B in BLCA. A. Comprehensive analysis of [file 13148_2026_2079_MOESM1_ESM.zip › Supplementary files/figS4.tif]

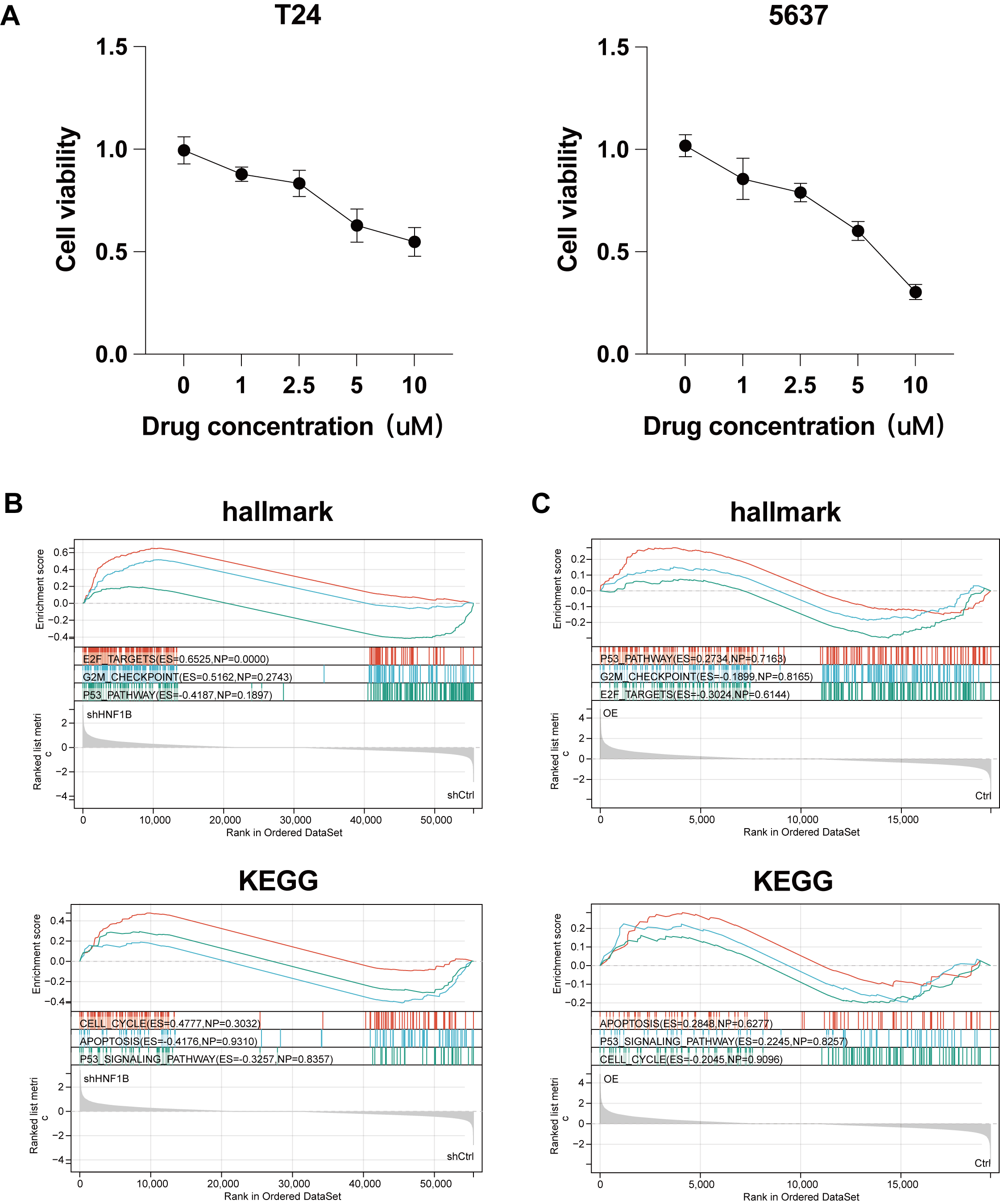

Supplement: Supplementary file 1 — Supplementary Fig. 1. Supplementary analysis of HNF1B in BLCA. A. Kaplan–Meier analysis of OS in the Xiangya cohort stratified by high vs. low HNF1B expression. B. Comparison of HNF1B mRNA levels between tumor and adjacent normal tissues in the TCGA-BLCA cohort. C. Representative IHC images of HNF1B in BLCA and normal urothelial tissues from the HPA. ns, not significant. Supplementary Fig. 2. Control experiments for HNF1B function in BLCA cells. A. Validation of HNF1B knockdown (shHNF1B-3 vs. shCtrl) and overexpression (OE vs. Ctrl) efficiency by qRT-PCR and Western blot. B. Cell proliferation measured by CCK-8 assay following HNF1B knockdown or overexpression. C, D. Clonogenic survival assessed by colony formation assay following HNF1B knockdown in 5637 cells (C) or overexpression in T24 cells (D). E. Effects of HNF1B knockdown or overexpression on cell migration, assessed by wound healing assays. F, G. Effects of HNF1B knockdown in 5637 cells (F) or overexpression in T24 cells (G) on cell migration and invasion, assessed by Transwell assays. ns, not significant, * P < 0.05, ** P < 0.01, *** P < 0.001, **** P < 0.0001. Supplementary Fig. 3. Comprehensive analysis of HNF1B genetic and epigenetic alterations in BLCA. A. Correlation between HNF1B expression and its promoter methylation at site cg19378036 in the TCGA-BLCA cohort. B. Analysis of HNF1B single nucleotide polymorphisms in the TCGA-BLCA cohort. C. Correlation between HNF1B expression and its CNV in the TCGA-BLCA cohort. D. DNA methylation across all HNF1B CpG sites in tumor (Ca) vs. normal (Nor) tissues in the TCGA-BLCA cohort E. Comparison of DNA methylation at three specific HNF1B promoter CpG sites in tumor vs. normal tissues in the TCGA-BLCA cohort. F. HNF1B promoter methylation in three BLCA cell lines assessed by BSP. ns, not significant, * P < 0.05. Supplementary Fig. 4. Integrated analysis of transcriptomic alterations and genetic landscape associated with HNF1B in BLCA. A. Comprehensive analysis of [file 13148_2026_2079_MOESM1_ESM.zip › Supplementary files/figS5.tif]

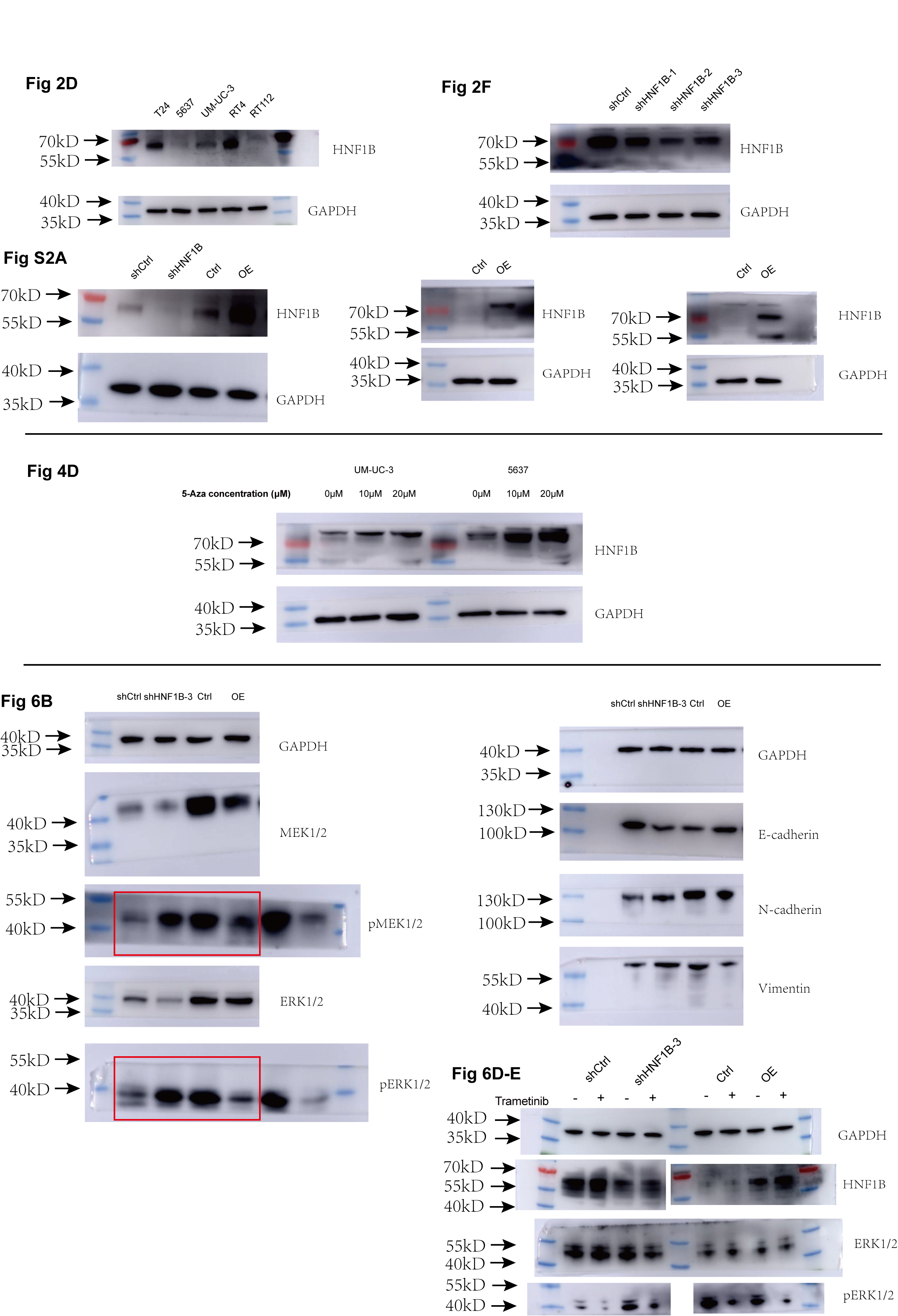

Supplement: Supplementary file 1 — Supplementary Fig. 1. Supplementary analysis of HNF1B in BLCA. A. Kaplan–Meier analysis of OS in the Xiangya cohort stratified by high vs. low HNF1B expression. B. Comparison of HNF1B mRNA levels between tumor and adjacent normal tissues in the TCGA-BLCA cohort. C. Representative IHC images of HNF1B in BLCA and normal urothelial tissues from the HPA. ns, not significant. Supplementary Fig. 2. Control experiments for HNF1B function in BLCA cells. A. Validation of HNF1B knockdown (shHNF1B-3 vs. shCtrl) and overexpression (OE vs. Ctrl) efficiency by qRT-PCR and Western blot. B. Cell proliferation measured by CCK-8 assay following HNF1B knockdown or overexpression. C, D. Clonogenic survival assessed by colony formation assay following HNF1B knockdown in 5637 cells (C) or overexpression in T24 cells (D). E. Effects of HNF1B knockdown or overexpression on cell migration, assessed by wound healing assays. F, G. Effects of HNF1B knockdown in 5637 cells (F) or overexpression in T24 cells (G) on cell migration and invasion, assessed by Transwell assays. ns, not significant, * P < 0.05, ** P < 0.01, *** P < 0.001, **** P < 0.0001. Supplementary Fig. 3. Comprehensive analysis of HNF1B genetic and epigenetic alterations in BLCA. A. Correlation between HNF1B expression and its promoter methylation at site cg19378036 in the TCGA-BLCA cohort. B. Analysis of HNF1B single nucleotide polymorphisms in the TCGA-BLCA cohort. C. Correlation between HNF1B expression and its CNV in the TCGA-BLCA cohort. D. DNA methylation across all HNF1B CpG sites in tumor (Ca) vs. normal (Nor) tissues in the TCGA-BLCA cohort E. Comparison of DNA methylation at three specific HNF1B promoter CpG sites in tumor vs. normal tissues in the TCGA-BLCA cohort. F. HNF1B promoter methylation in three BLCA cell lines assessed by BSP. ns, not significant, * P < 0.05. Supplementary Fig. 4. Integrated analysis of transcriptomic alterations and genetic landscape associated with HNF1B in BLCA. A. Comprehensive analysis of [file 13148_2026_2079_MOESM1_ESM.zip › Supplementary files/Original image of western blot experiments.tif]
